# Poly[[hexa­aqua­sesqui(μ-benzene-1,2,4,5-tetra­carboxyl­ato)dicopper(II)disodium] monohydrate]

**DOI:** 10.1107/S1600536814014755

**Published:** 2014-07-02

**Authors:** Magatte Camara, Mohamed Fadel Keita, Cherif Cheikh Samsidine Cisse, Carole Daiguebonne, Olivier Guillou

**Affiliations:** a Université Assane Seck de Ziguinchor, LCPM-Groupe Materiaux Inorganiques: Chimie Douce et Cristallographie, BP 523 Ziguinchor, Senegal; b INSA, UMR 6226 "Institut des Sciences Chimiques de Rennes", F-35708 Rennes, France

**Keywords:** crystal structure

## Abstract

In the title compound, {[Cu_2_Na_2_(C_10_H_2_O_8_)_1.5_(H_2_O)_6_]·H_2_O}_*n*_, the Cu^2+^ ion is hexa­coordinated by five O atoms from benzene-1,2,4,5-tetra­carboxyl­ate (btec^4−^) ligands and one water mol­ecule. The Na^+^ ion is also hexa­coordinated, by four O atoms from btec^4−^ ligands and two water mol­ecules. One of the two btec^4−^ mol­ecules sits on a crystallographic inversion centre. CuO_6_ and NaO_6_ octa­hedra are connected, forming bi-dimensional layers. These layers, which extend parallel to the *ac* plane, are further inter­connected by μ_10_- or μ_11_-bridging btec^4−^ ligands and by O—H⋯O hydrogen bonds, involving both btec^4−^ ligands and water mol­ecules, forming a three-dimensional network.

## Related literature   

For related structures, see: Camara *et al.* (2013 ▶); Luo *et al.* (2013 ▶); Gong & Zhang (2011 ▶); Liu *et al.* (2010 ▶); Zhang *et al.* (2007 ▶). For related crystal-growth methods in gels, see: Henisch (1988 ▶); Henisch & Roy (1970 ▶); Daiguebonne *et al.* (2003 ▶). 
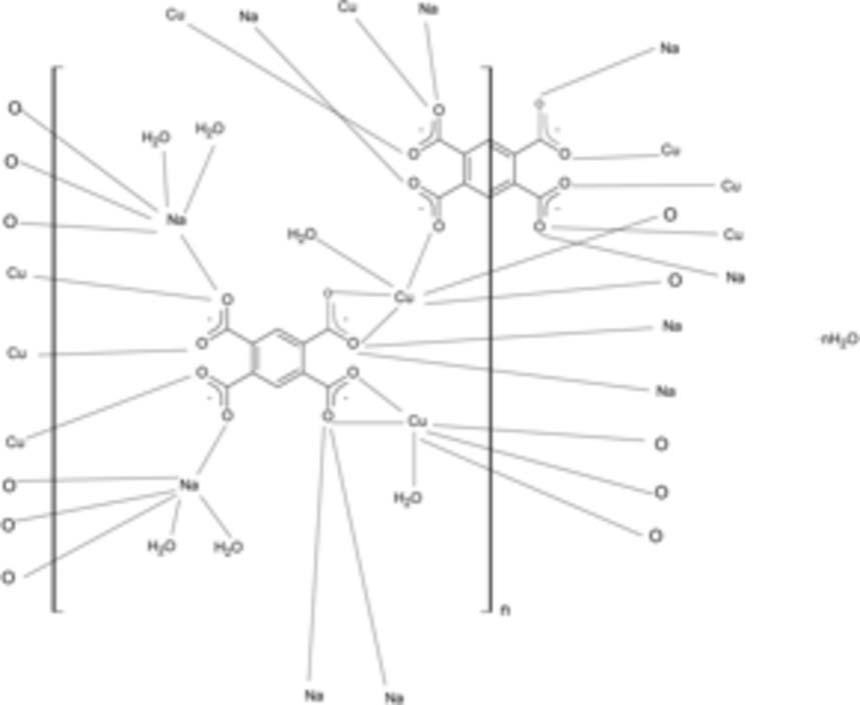


## Experimental   

### 

#### Crystal data  

[Cu_2_Na_2_(C_10_H_2_O_8_)_1.5_(H_2_O)_6_]·H_2_O
*M* *_r_* = 674.34
Monoclinic, 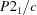

*a* = 8.0844 (1) Å
*b* = 16.9103 (3) Å
*c* = 15.6815 (3) Å
β = 98.055 (1)°
*V* = 2122.66 (6) Å^3^
*Z* = 4
Mo *K*α radiation
μ = 2.15 mm^−1^
*T* = 293 K
0.11 × 0.08 × 0.07 mm

#### Data collection  

Bruker APEXII diffractometer
Absorption correction: multi-scan (*SADABS*; Bruker, 2007 ▶) *T*_min_ = 0.701, *T*_max_ = 0.848
34613 measured reflections
8438 independent reflections
4356 reflections with *I* > 2σ(*I*)
*R*_int_ = 0.058

#### Refinement  

*R*[*F* ^2^ > 2σ(*F* ^2^)] = 0.049
*wR*(*F* ^2^) = 0.144
*S* = 0.94
8438 reflections
373 parameters
15 restraints
H atoms treated by a mixture of independent and constrained refinement
Δρ_max_ = 1.49 e Å^−3^
Δρ_min_ = −1.02 e Å^−3^

### 

Data collection: *APEX2* (Bruker, 2007 ▶); cell refinement: *SAINT* (Bruker, 2007 ▶); data reduction: *SAINT*; program(s) used to solve structure: *SHELXS97* (Sheldrick, 2008 ▶); program(s) used to refine structure: *SHELXL2014* (Sheldrick, 2008 ▶); molecular graphics: *DIAMOND* (Brandenburg, 1999 ▶); software used to prepare material for publication: *publCIF* (Westrip, 2010 ▶).

## Supplementary Material

Crystal structure: contains datablock(s) I. DOI: 10.1107/S1600536814014755/pk2525sup1.cif

Structure factors: contains datablock(s) I. DOI: 10.1107/S1600536814014755/pk2525Isup2.hkl

CCDC reference: 1009732

Additional supporting information:  crystallographic information; 3D view; checkCIF report

## Figures and Tables

**Table 1:** Hydrogen-bond geometry (Å, °)

*D*—H⋯*A*	*D*—H	H⋯*A*	*D*⋯*A*	*D*—H⋯*A*
O4—H42⋯O112^i^	0.81 (2)	1.93 (2)	2.737 (3)	174 (4)
O2—H22⋯O511	0.82 (2)	1.91 (2)	2.697 (3)	162 (4)
O1—H11⋯O812	0.80 (2)	1.95 (2)	2.708 (3)	159 (4)
O2—H21⋯O*W*1^ii^	0.78 (2)	1.85 (2)	2.618 (3)	167 (4)
O5—H51⋯O512^iii^	0.87 (2)	1.91 (2)	2.744 (3)	161 (4)
O1—H12⋯O2^iv^	0.81 (2)	1.99 (2)	2.794 (3)	176 (4)
O3—H32⋯O1^v^	0.85 (2)	2.36 (3)	2.977 (3)	130 (3)

Symmetry codes: (i) 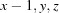; (ii) 
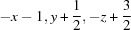
; (iii) 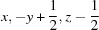; (iv) 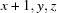; (v) 
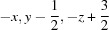
.

**Fig. 1.**
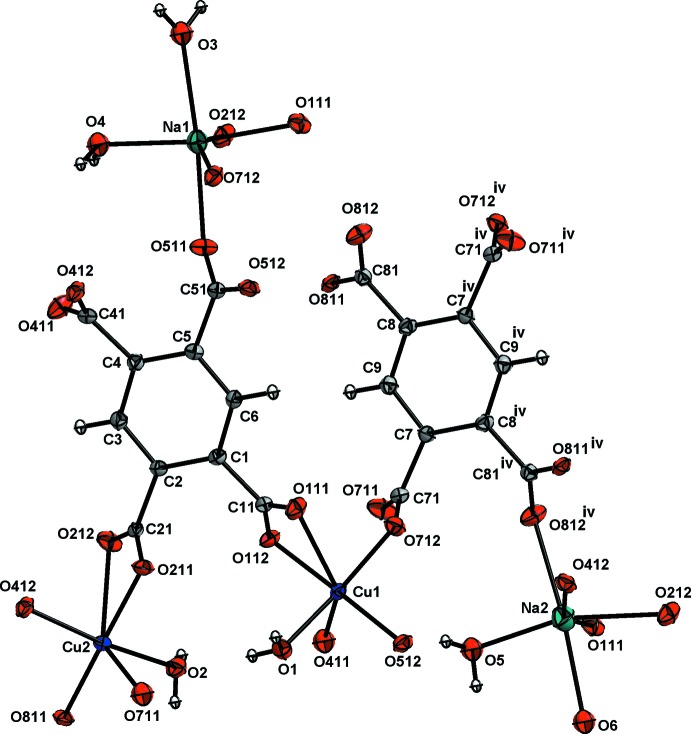
Extended asymetric unit of the title compound, with the atom-numbering scheme. Displacement ellipsoids are drawn at the 50% probability level. [Symmetry code: (iv) x, -y + 1/2, z - 1/2.]

**Fig. 2.**
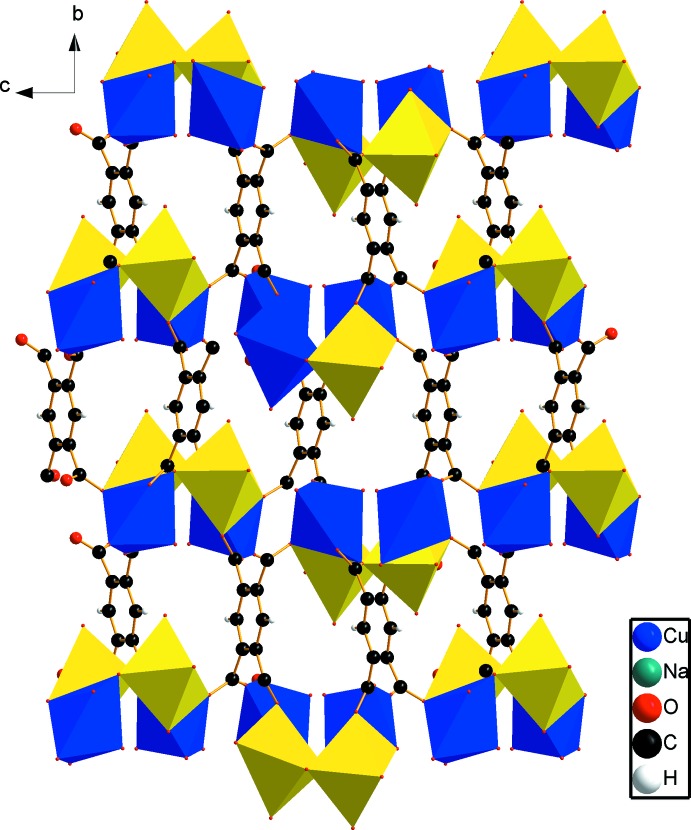
A view of the three-dimensional network of the title compound, projected perpendicular to the bc-plane.

## supplementary crystallographic information

### S1. Comment

In recent years, much attention has been paid to coordination polymers that involve covalent bonds or supra­molecular contacts. A huge number of novel compounds with inter­esting crystal structures and topologies have been reported. As part of this research, benzene-1,2,4,5-tetra­carboxyl­ate (btec^4-^) can be used as a ligand to form various supra­molecular architectures through its four rigid carboxyl groups [*see* Camara *et al.* (2013), Luo *et al.* (2013), Gong & Zhang (2011), Liu *et al.* (2010), Zhang *et al.* (2007)]. In order to enrich this family of compounds, we recently undertook a study devoted to reactions of H_4_btec with metal ions in gel media. In particular, we were inter­ested in such reactions with agar-agar gel bridges in U-shaped tubes, and we have successfully synthesized a novel polymeric complex from H_4_btec and copper (II) chloride. We report here the synthesis and the crystal structure of the title coordination polymer. As shown in Fig. 1, the asymmetric unit of the title compound contains two Cu(II) ions, two Na(I) ions, coordinated water molecules and three btec^4-^ ligands. One of the two crystallographically distinct btec^4-^ ligands is located on an inversion centre. One btec^4-^ ligand acts as a m_10_-bridge that links six Cu(II) ions and four Na(I) ions, while the other acts as a m_11_-bridge that links five Cu(II) ions and six Na(I) ions. Cu(II) ions are hexa-coordinated by five O atoms from four btec^4-^ ligands and one O atom from a water molecule, while Na(I) ions are hexa-coordinated by four O atoms from four btec^4-^ ligands and two O atoms from two water molecules. As shown in Fig. 2, two-dimensional Cu–O–Na layers extend parallel to the ac plane. Furthermore, these two-dimensional layers are inter­connected by m_10_- or m_11_-bridging btec^4-^ ligands and by O—H···O hydrogen bonds, forming a three-dimensional framework.

### S2. Experimental

All reagents were used as obtained without further purification. Copper (II) chloride was purchased from STREM Chemicals. 1,2,4,5-benzene­tetra­carb­oxy­lic acid was purchased from Acros Organics. Its sodium salt was prepared by addition of four equivalents of sodium hydroxide to a suspension of 1,2,4,5-benzene­tetra­carb­oxy­lic acid in de-ionized water until complete dissolution. Then, the solution was evaporated to dryness. The solid phase was then put in suspension in ethanol, stirred and refluxed for 1 h. After filtration and drying in a desiccator, a white powder of tetra-sodium 1,2,4,5-benzene-tetra-carboxyl­ate was obtained (yield = 90%). Single crystals of the coordination polymer were obtained by slow diffusion of dilute aqueous solutions of Cu(II) chloride (0.25 mmol in 20mL) and of the sodium salt of 1,2,4,5-benzene­tetra-carb­oxy­lic acid (0.25 mmol in 20mL) through agar-agar gel bridges in U-shaped tubes. The gel was purchased from Acros Organics and gelled according to established procedure [see for example, Henisch *et al.* (1988), Henisch *et al.* (1970), Daiguebonne *et al.* (2003)]. After several weeks, colorless single crystals were obtained.

### S3. Refinement

The H-atoms from water molecules that could be found in difference maps were included with constrained coordinates. Some hydrogen atoms could not be reliably located, so these have only been taken into account in the formula (*i.e.* not in the model).

### Crystal data

**Table:**

[Cu_2_Na_2_(C_10_H_2_O_8_)_1.5_(H_2_O)_6_]·H_2_O	*F*(000) = 1356
*M**_r_* = 674.34	*D*_x_ = 2.110 Mg m^−^^3^
Monoclinic, *P*2_1_/*c*	Mo *K*α radiation, λ = 0.71073 Å
*a* = 8.0844 (1) Å	Cell parameters from 31092 reflections
*b* = 16.9103 (3) Å	θ = 2.9–32.0°
*c* = 15.6815 (3) Å	µ = 2.15 mm^−^^1^
β = 98.055 (1)°	*T* = 293 K
*V* = 2122.66 (6) Å^3^	Cobblestone, blue
*Z* = 4	0.11 × 0.08 × 0.07 mm

### Data collection

**Table:**

Bruker APEXII diffractometer	8438 independent reflections
Radiation source: Fine-focus sealed tube	4356 reflections with *I* > 2σ(*I*)
Graphite monochromator	*R*_int_ = 0.058
CCD rotation images, thin slices scans	θ_max_ = 34.8°, θ_min_ = 3.5°
Absorption correction: multi-scan (*SADABS*; Bruker, 2007)	*h* = −11→12
*T*_min_ = 0.701, *T*_max_ = 0.848	*k* = −26→21
34613 measured reflections	*l* = −20→24

### Refinement

**Table:**

Refinement on *F*^2^	15 restraints
Least-squares matrix: full	Hydrogen site location: inferred from neighbouring sites
*R*[*F*^2^ > 2σ(*F*^2^)] = 0.049	H atoms treated by a mixture of independent and constrained refinement
*wR*(*F*^2^) = 0.144	*w* = 1/[σ^2^(*F*_o_^2^) + (0.0813*P*)^2^] where *P* = (*F*_o_^2^ + 2*F*_c_^2^)/3
*S* = 0.94	(Δ/σ)_max_ = 0.010
8438 reflections	Δρ_max_ = 1.49 e Å^−^^3^
373 parameters	Δρ_min_ = −1.02 e Å^−^^3^

### Special details

**Table:**

Geometry. All e.s.d.'s (except the e.s.d. in the dihedral angle between two l.s. planes) are estimated using the full covariance matrix. The cell e.s.d.'s are taken into account individually in the estimation of e.s.d.'s in distances, angles and torsion angles; correlations between e.s.d.'s in cell parameters are only used when they are defined by crystal symmetry. An approximate (isotropic) treatment of cell e.s.d.'s is used for estimating e.s.d.'s involving l.s. planes.

### Fractional atomic coordinates and isotropic or equivalent isotropic displacement parameters (Å^2^)

**Table:**

	*x*	*y*	*z*	*U*_iso_*/*U*_eq_	
Cu1	0.15805 (4)	0.25569 (2)	0.80720 (2)	0.01609 (10)	
Cu2	−0.45959 (4)	0.26469 (2)	0.54858 (2)	0.01697 (10)	
Na1	−0.17322 (14)	0.13536 (7)	0.78727 (8)	0.0284 (3)	
Na2	−0.13005 (15)	0.15430 (8)	0.56592 (9)	0.0329 (3)	
O112	0.2924 (2)	0.17014 (11)	0.76659 (13)	0.0195 (4)	
O211	−0.5911 (2)	0.17609 (11)	0.58371 (13)	0.0215 (4)	
O1	0.1863 (2)	0.31650 (12)	0.69877 (13)	0.0224 (4)	
O4	−0.4112 (3)	0.11473 (14)	0.85120 (16)	0.0316 (5)	
O511	−0.2307 (2)	0.27440 (11)	0.78365 (14)	0.0256 (5)	
O811	−0.3190 (2)	0.35241 (11)	0.52142 (13)	0.0204 (4)	
O812	−0.0757 (2)	0.29573 (12)	0.57293 (14)	0.0271 (5)	
O111	0.0408 (2)	0.13710 (12)	0.70094 (13)	0.0230 (4)	
O212	−0.3474 (2)	0.13420 (12)	0.65142 (14)	0.0260 (5)	
O2	−0.4857 (2)	0.31465 (12)	0.66155 (14)	0.0228 (4)	
O412	−0.3727 (2)	0.19907 (11)	0.46253 (13)	0.0215 (4)	
O711	0.3056 (2)	0.15899 (12)	0.98797 (15)	0.0308 (5)	
O512	0.0052 (2)	0.33892 (11)	0.83143 (13)	0.0214 (4)	
O411	0.3855 (2)	0.31657 (12)	0.87600 (15)	0.0317 (5)	
C8	−0.0749 (3)	0.42790 (15)	0.51646 (17)	0.0166 (5)	
O712	0.0771 (2)	0.18442 (11)	0.89280 (13)	0.0222 (4)	
C5	−0.2469 (3)	0.40780 (15)	0.82911 (17)	0.0172 (5)	
C81	−0.1595 (3)	0.35232 (15)	0.53885 (18)	0.0177 (5)	
C51	−0.1527 (3)	0.33362 (15)	0.81388 (18)	0.0165 (5)	
C7	0.0755 (3)	0.07233 (15)	0.98119 (17)	0.0167 (5)	
C6	−0.1850 (3)	0.47968 (15)	0.80321 (19)	0.0191 (6)	
H6	−0.0866	0.4800	0.7788	0.023*	
O5	0.1040 (3)	0.13627 (18)	0.50399 (18)	0.0505 (7)	
C4	−0.3963 (3)	0.09269 (15)	0.36547 (17)	0.0159 (5)	
O3	−0.1803 (4)	−0.00918 (16)	0.7752 (2)	0.0556 (8)	
OW1	−0.4788 (3)	−0.03675 (15)	0.88423 (19)	0.0526 (7)	
C2	−0.5855 (3)	0.05086 (15)	0.64934 (18)	0.0161 (5)	
C21	−0.4989 (3)	0.12554 (15)	0.62866 (17)	0.0160 (5)	
C1	0.2666 (3)	0.05056 (15)	0.68709 (18)	0.0169 (5)	
C9	0.1473 (3)	−0.00066 (16)	0.96511 (18)	0.0185 (5)	
H9	0.2465	−0.0013	0.9414	0.022*	
C11	0.1920 (3)	0.12402 (15)	0.71862 (17)	0.0166 (5)	
C3	−0.4787 (3)	0.02144 (16)	0.37516 (18)	0.0194 (6)	
H3	−0.5785	0.0219	0.3984	0.023*	
C41	−0.4697 (3)	0.16482 (15)	0.40121 (17)	0.0159 (5)	
O6	−0.1540 (4)	0.0144 (2)	0.5561 (3)	0.0822 (11)	
C71	0.1614 (3)	0.14565 (15)	0.95356 (18)	0.0179 (6)	
H42	−0.494 (3)	0.133 (2)	0.824 (2)	0.050*	
H22	−0.425 (4)	0.300 (2)	0.7052 (17)	0.050*	
H11	0.122 (3)	0.300 (2)	0.6598 (18)	0.050*	
H21	−0.481 (4)	0.3594 (12)	0.650 (2)	0.050*	
H51	0.088 (5)	0.153 (2)	0.4512 (14)	0.050*	
H41	−0.396 (4)	0.136 (2)	0.8981 (15)	0.050*	
H52	0.104 (5)	0.0878 (10)	0.499 (2)	0.050*	
H31	−0.176 (5)	−0.039 (2)	0.7328 (16)	0.050*	
H12	0.282 (2)	0.318 (2)	0.690 (2)	0.050*	
H32	−0.166 (5)	−0.0448 (19)	0.8137 (18)	0.050*	

### Atomic displacement parameters (Å^2^)

**Table:**

	*U*^11^	*U*^22^	*U*^33^	*U*^12^	*U*^13^	*U*^23^
Cu1	0.01508 (17)	0.01221 (17)	0.0216 (2)	0.00191 (11)	0.00483 (13)	−0.00013 (12)
Cu2	0.01881 (18)	0.01265 (17)	0.0207 (2)	−0.00348 (11)	0.00713 (13)	−0.00177 (13)
Na1	0.0240 (6)	0.0267 (7)	0.0351 (7)	−0.0005 (5)	0.0067 (5)	0.0006 (5)
Na2	0.0270 (6)	0.0356 (7)	0.0363 (8)	0.0007 (5)	0.0054 (5)	0.0006 (6)
O112	0.0192 (9)	0.0127 (9)	0.0264 (11)	0.0019 (7)	0.0021 (8)	−0.0046 (8)
O211	0.0209 (10)	0.0132 (9)	0.0307 (12)	−0.0032 (7)	0.0047 (8)	0.0029 (8)
O1	0.0197 (10)	0.0255 (11)	0.0226 (11)	0.0017 (8)	0.0057 (8)	0.0037 (9)
O4	0.0267 (12)	0.0390 (14)	0.0286 (13)	0.0027 (9)	0.0022 (9)	−0.0038 (10)
O511	0.0274 (11)	0.0149 (10)	0.0328 (13)	0.0023 (8)	−0.0015 (9)	−0.0042 (8)
O811	0.0189 (10)	0.0136 (9)	0.0297 (12)	−0.0050 (7)	0.0063 (8)	0.0008 (8)
O812	0.0270 (11)	0.0190 (11)	0.0338 (13)	−0.0015 (8)	−0.0015 (9)	0.0063 (9)
O111	0.0161 (10)	0.0230 (11)	0.0296 (12)	0.0032 (7)	0.0020 (8)	−0.0030 (8)
O212	0.0175 (10)	0.0261 (11)	0.0338 (13)	−0.0070 (8)	0.0015 (8)	0.0023 (9)
O2	0.0277 (11)	0.0203 (10)	0.0203 (12)	−0.0015 (8)	0.0034 (8)	−0.0017 (9)
O412	0.0198 (9)	0.0197 (10)	0.0255 (11)	−0.0020 (7)	0.0050 (8)	−0.0092 (8)
O711	0.0218 (11)	0.0276 (12)	0.0405 (14)	−0.0103 (8)	−0.0042 (9)	0.0035 (10)
O512	0.0171 (10)	0.0147 (10)	0.0330 (12)	0.0031 (7)	0.0058 (8)	−0.0023 (8)
O411	0.0262 (11)	0.0282 (12)	0.0390 (14)	−0.0121 (9)	−0.0016 (9)	0.0025 (10)
C8	0.0179 (13)	0.0145 (13)	0.0174 (14)	−0.0042 (9)	0.0030 (10)	−0.0013 (10)
O712	0.0223 (10)	0.0199 (10)	0.0246 (11)	−0.0003 (8)	0.0041 (8)	0.0056 (8)
C5	0.0199 (13)	0.0132 (13)	0.0193 (14)	0.0023 (9)	0.0054 (10)	−0.0002 (10)
C81	0.0181 (13)	0.0167 (13)	0.0194 (14)	−0.0027 (10)	0.0064 (10)	−0.0046 (10)
C51	0.0186 (13)	0.0138 (13)	0.0179 (14)	0.0020 (9)	0.0054 (10)	0.0009 (10)
C7	0.0145 (12)	0.0146 (13)	0.0213 (15)	0.0004 (9)	0.0039 (10)	0.0033 (10)
C6	0.0184 (13)	0.0157 (13)	0.0251 (16)	0.0001 (10)	0.0096 (11)	0.0026 (11)
O5	0.0407 (15)	0.075 (2)	0.0365 (16)	−0.0024 (15)	0.0076 (12)	0.0115 (15)
C4	0.0162 (12)	0.0153 (13)	0.0167 (14)	−0.0008 (9)	0.0038 (10)	−0.0028 (10)
O3	0.0532 (16)	0.0305 (15)	0.087 (2)	0.0089 (12)	0.0230 (17)	0.0051 (15)
OW1	0.0622 (17)	0.0350 (15)	0.0592 (19)	−0.0121 (12)	0.0036 (14)	−0.0014 (13)
C2	0.0168 (12)	0.0116 (12)	0.0203 (14)	−0.0009 (9)	0.0045 (10)	0.0001 (10)
C21	0.0177 (13)	0.0132 (12)	0.0188 (14)	−0.0041 (9)	0.0082 (10)	−0.0055 (10)
C1	0.0184 (13)	0.0131 (12)	0.0200 (14)	−0.0001 (9)	0.0049 (10)	−0.0026 (10)
C9	0.0160 (13)	0.0173 (13)	0.0227 (15)	0.0010 (10)	0.0050 (10)	0.0021 (11)
C11	0.0217 (14)	0.0110 (12)	0.0185 (14)	0.0004 (10)	0.0076 (10)	−0.0002 (10)
C3	0.0172 (13)	0.0194 (14)	0.0228 (15)	−0.0013 (10)	0.0074 (11)	−0.0037 (11)
C41	0.0181 (13)	0.0113 (12)	0.0186 (14)	−0.0013 (9)	0.0036 (10)	0.0006 (10)
O6	0.068 (2)	0.073 (2)	0.106 (3)	−0.0037 (17)	0.015 (2)	−0.015 (2)
C71	0.0219 (14)	0.0115 (12)	0.0217 (15)	0.0003 (10)	0.0077 (11)	0.0000 (10)

### Geometric parameters (Å, º)

**Table:**

Cu1—O512	1.9450 (17)	O111—C11	1.235 (3)
Cu1—O112	1.9676 (17)	O212—C21	1.235 (3)
Cu1—O712	1.9821 (19)	O412—C41	1.290 (3)
Cu1—O1	2.027 (2)	O711—C71	1.235 (3)
Cu1—O411	2.247 (2)	O711—Cu2^ii^	2.380 (2)
Cu1—Na1	3.3431 (12)	O512—C51	1.271 (3)
Cu2—O412	1.9523 (18)	O411—C41^ii^	1.223 (3)
Cu2—O811	1.9519 (17)	C8—C9^iii^	1.390 (4)
Cu2—O211	1.9603 (18)	C8—C7^iv^	1.404 (3)
Cu2—O2	2.000 (2)	C8—C81	1.514 (3)
Cu2—O711^i^	2.380 (2)	O712—C71	1.273 (3)
Cu2—Na2	3.2331 (13)	C5—C6	1.396 (4)
Na1—O4	2.317 (2)	C5—C4^v^	1.406 (3)
Na1—O111	2.342 (2)	C5—C51	1.504 (3)
Na1—O212	2.384 (2)	C7—C8^v^	1.404 (3)
Na1—O511	2.396 (2)	C7—C9	1.402 (4)
Na1—O3	2.452 (3)	C7—C71	1.514 (3)
Na1—O712	2.566 (2)	C6—C1^iii^	1.387 (4)
Na1—Na2	3.5514 (19)	C4—C3	1.396 (4)
Na2—O5	2.266 (3)	C4—C5^iv^	1.406 (3)
Na2—O6	2.377 (4)	C4—C41	1.499 (3)
Na2—O212	2.379 (2)	C2—C3^vi^	1.403 (3)
Na2—O111	2.378 (2)	C2—C1^vii^	1.407 (4)
Na2—O812	2.431 (2)	C2—C21	1.501 (3)
Na2—O412	2.482 (2)	C1—C6^viii^	1.387 (4)
O112—C11	1.289 (3)	C1—C2^ix^	1.407 (4)
O211—C21	1.279 (3)	C1—C11	1.495 (3)
O511—C51	1.241 (3)	C9—C8^viii^	1.390 (4)
O811—C81	1.280 (3)	C3—C2^vi^	1.403 (3)
O812—C81	1.248 (3)	C41—O411^i^	1.223 (3)
			
O512—Cu1—O112	171.78 (8)	O5—Na2—Cu2	140.32 (9)
O512—Cu1—O712	91.64 (8)	O6—Na2—Cu2	120.71 (9)
O112—Cu1—O712	91.24 (8)	O212—Na2—Cu2	57.65 (5)
O512—Cu1—O1	86.91 (8)	O111—Na2—Cu2	120.96 (6)
O112—Cu1—O1	88.24 (8)	O812—Na2—Cu2	65.00 (5)
O712—Cu1—O1	164.40 (8)	O412—Na2—Cu2	37.09 (4)
O512—Cu1—O411	94.25 (8)	O5—Na2—Na1	127.53 (9)
O112—Cu1—O411	92.33 (8)	O6—Na2—Na1	87.46 (10)
O712—Cu1—O411	106.20 (8)	O212—Na2—Na1	41.83 (6)
O1—Cu1—O411	89.40 (8)	O111—Na2—Na1	40.82 (5)
O512—Cu1—Na1	86.34 (6)	O812—Na2—Na1	94.95 (6)
O112—Cu1—Na1	89.62 (6)	O412—Na2—Na1	119.28 (6)
O712—Cu1—Na1	49.99 (6)	Cu2—Na2—Na1	86.65 (3)
O1—Cu1—Na1	114.41 (6)	C11—O112—Cu1	107.64 (15)
O411—Cu1—Na1	156.16 (6)	C21—O211—Cu2	111.63 (16)
O412—Cu2—O811	90.24 (8)	C51—O511—Na1	134.11 (18)
O412—Cu2—O211	91.24 (8)	C81—O811—Cu2	123.53 (17)
O811—Cu2—O211	176.00 (8)	C81—O812—Na2	130.61 (18)
O412—Cu2—O2	160.91 (9)	C11—O111—Na1	131.02 (19)
O811—Cu2—O2	90.52 (8)	C11—O111—Na2	130.92 (18)
O211—Cu2—O2	86.87 (8)	Na1—O111—Na2	97.59 (8)
O412—Cu2—O711^i^	112.09 (8)	C21—O212—Na2	129.39 (19)
O811—Cu2—O711^i^	87.53 (8)	C21—O212—Na1	134.18 (18)
O211—Cu2—O711^i^	95.34 (8)	Na2—O212—Na1	96.43 (8)
O2—Cu2—O711^i^	86.99 (8)	C41—O412—Cu2	122.04 (16)
O412—Cu2—Na2	50.05 (6)	C41—O412—Na2	133.81 (17)
O811—Cu2—Na2	87.65 (6)	Cu2—O412—Na2	92.86 (8)
O211—Cu2—Na2	90.46 (6)	C71—O711—Cu2^ii^	157.62 (19)
O2—Cu2—Na2	110.93 (6)	C51—O512—Cu1	123.57 (17)
O711^i^—Cu2—Na2	161.47 (6)	C41^ii^—O411—Cu1	162.7 (2)
O4—Na1—O111	167.85 (10)	C9^iii^—C8—C7^iv^	119.8 (2)
O4—Na1—O212	87.88 (9)	C9^iii^—C8—C81	117.9 (2)
O111—Na1—O212	82.84 (8)	C7^iv^—C8—C81	122.2 (2)
O4—Na1—O511	89.31 (9)	C71—O712—Cu1	128.85 (17)
O111—Na1—O511	97.50 (8)	C71—O712—Na1	127.40 (17)
O212—Na1—O511	84.18 (8)	Cu1—O712—Na1	93.74 (8)
O4—Na1—O3	82.61 (10)	C6—C5—C4^v^	119.1 (2)
O111—Na1—O3	88.80 (9)	C6—C5—C51	118.0 (2)
O212—Na1—O3	85.32 (11)	C4^v^—C5—C51	122.8 (2)
O511—Na1—O3	166.98 (10)	O812—C81—O811	124.2 (2)
O4—Na1—O712	113.41 (9)	O812—C81—C8	120.7 (2)
O111—Na1—O712	77.81 (7)	O811—C81—C8	115.1 (2)
O212—Na1—O712	153.24 (8)	O511—C51—O512	125.5 (2)
O511—Na1—O712	80.26 (7)	O511—C51—C5	119.5 (2)
O3—Na1—O712	112.32 (10)	O512—C51—C5	115.0 (2)
O4—Na1—Cu1	138.94 (8)	C8^v^—C7—C9	118.1 (2)
O111—Na1—Cu1	53.08 (5)	C8^v^—C7—C71	124.9 (2)
O212—Na1—Cu1	116.99 (6)	C9—C7—C71	116.8 (2)
O511—Na1—Cu1	63.58 (5)	C1^iii^—C6—C5	121.7 (2)
O3—Na1—Cu1	128.59 (8)	C3—C4—C5^iv^	119.3 (2)
O712—Na1—Cu1	36.27 (4)	C3—C4—C41	116.2 (2)
O4—Na1—Na2	129.62 (8)	C5^iv^—C4—C41	124.3 (2)
O111—Na1—Na2	41.59 (6)	C3^vi^—C2—C1^vii^	118.8 (2)
O212—Na1—Na2	41.74 (5)	C3^vi^—C2—C21	118.2 (2)
O511—Na1—Na2	86.20 (7)	C1^vii^—C2—C21	122.9 (2)
O3—Na1—Na2	91.09 (9)	O212—C21—O211	123.9 (2)
O712—Na1—Na2	115.12 (6)	O212—C21—C2	120.7 (2)
Cu1—Na1—Na2	81.34 (3)	O211—C21—C2	115.3 (2)
O5—Na2—O6	84.46 (12)	C6^viii^—C1—C2^ix^	119.6 (2)
O5—Na2—O212	161.73 (11)	C6^viii^—C1—C11	117.5 (2)
O6—Na2—O212	80.49 (11)	C2^ix^—C1—C11	122.8 (2)
O5—Na2—O111	87.09 (9)	C8^viii^—C9—C7	122.0 (2)
O6—Na2—O111	88.17 (12)	O111—C11—O112	123.0 (2)
O212—Na2—O111	82.16 (8)	O111—C11—C1	120.5 (2)
O5—Na2—O812	89.73 (10)	O112—C11—C1	116.5 (2)
O6—Na2—O812	174.00 (11)	C2^vi^—C3—C4	121.5 (2)
O212—Na2—O812	104.95 (8)	O411^i^—C41—O412	125.4 (2)
O111—Na2—O812	90.07 (8)	O411^i^—C41—C4	120.0 (2)
O5—Na2—O412	113.10 (10)	O412—C41—C4	114.4 (2)
O6—Na2—O412	102.21 (11)	O711—C71—O712	127.3 (2)
O212—Na2—O412	80.39 (7)	O711—C71—C7	117.9 (2)
O111—Na2—O412	157.86 (8)	O712—C71—C7	114.7 (2)
O812—Na2—O412	81.41 (7)		

Symmetry codes: (i) *x*−1, −*y*+1/2, *z*−1/2; (ii) *x*+1, −*y*+1/2, *z*+1/2; (iii) −*x*, *y*+1/2, −*z*+3/2; (iv) *x*, −*y*+1/2, *z*−1/2; (v) *x*, −*y*+1/2, *z*+1/2; (vi) −*x*−1, −*y*, −*z*+1; (vii) *x*−1, *y*, *z*; (viii) −*x*, *y*−1/2, −*z*+3/2; (ix) *x*+1, *y*, *z*.

### Hydrogen-bond geometry (Å, º)

**Table:**

*D*—H···*A*	*D*—H	H···*A*	*D*···*A*	*D*—H···*A*
O4—H42···O112^vii^	0.81 (2)	1.93 (2)	2.737 (3)	174 (4)
O2—H22···O511	0.82 (2)	1.91 (2)	2.697 (3)	162 (4)
O1—H11···O812	0.80 (2)	1.95 (2)	2.708 (3)	159 (4)
O2—H21···O*W*1^x^	0.78 (2)	1.85 (2)	2.618 (3)	167 (4)
O5—H51···O512^iv^	0.87 (2)	1.91 (2)	2.744 (3)	161 (4)
O1—H12···O2^ix^	0.81 (2)	1.99 (2)	2.794 (3)	176 (4)
O3—H32···O1^viii^	0.85 (2)	2.36 (3)	2.977 (3)	130 (3)

Symmetry codes: (iv) *x*, −*y*+1/2, *z*−1/2; (vii) *x*−1, *y*, *z*; (viii) −*x*, *y*−1/2, −*z*+3/2; (ix) *x*+1, *y*, *z*; (x) −*x*−1, *y*+1/2, −*z*+3/2.
